# Alternative signaling network activation through different insulin receptor family members caused by pro-mitogenic antidiabetic insulin analogues in human mammary epithelial cells

**DOI:** 10.1186/s13058-015-0600-5

**Published:** 2015-07-19

**Authors:** Bas ter Braak, Steven Wink, Esmee Koedoot, Chantal Pont, Christine Siezen, Jan Willem van der Laan, Bob van de Water

**Affiliations:** Division of Toxicology, Leiden Academic Centre for Drug Research, Leiden University, Einsteinweg 55, Leiden, 2333 CC The Netherlands; Medicines Evaluation Board (MEB), Graadt van Roggenweg 500, Utrecht, 3531 AH The Netherlands; Centre for Health Protection, National Institute for Public Health and the Environment (RIVM), Antonie van Leeuwenhoeklaan 9, Bilthoven, 3721 MA The Netherlands

## Abstract

**Introduction:**

Insulin analogues are designed to have improved pharmacokinetic parameters compared to regular human insulin. This provides a sustained control of blood glucose levels in diabetic patients. All novel insulin analogues are tested for their mitogenic side effects, however these assays do not take into account the molecular mode of action of different insulin analogues. Insulin analogues can bind the insulin receptor and the insulin-like growth factor 1 receptor with different affinities and consequently will activate different downstream signaling pathways.

**Methods:**

Here we used a panel of MCF7 human breast cancer cell lines that selectively express either one of the isoforms of the INSR or the IGF1R. We applied a transcriptomics approach to assess the differential transcriptional programs activated in these cells by either insulin, IGF1 or X10 treatment.

**Results:**

Based on the differentially expressed genes between insulin versus IGF1 and X10 treatment, we retrieved a mitogenic classifier gene set. Validation by RT-qPCR confirmed the robustness of this gene set. The translational potential of these mitogenic classifier genes was examined in primary human mammary cells and in mammary gland tissue of mice in an in vivo model. The predictive power of the classifier genes was evaluated by testing all commercial insulin analogues in the in vitro model and defined X10 and glargine as the most potent mitogenic insulin analogues.

**Conclusions:**

We propose that these mitogenic classifier genes can be used to test the mitogenic potential of novel insulin analogues as well as other alternative molecules with an anticipated affinity for the IGF1R.

**Electronic supplementary material:**

The online version of this article (doi:10.1186/s13058-015-0600-5) contains supplementary material, which is available to authorized users.

## Introduction

Diabetes mellitus is the most common endocrine disease with over 380 million patients in 2013, worldwide [1]. A common treatment for both type-1 and type-2 diabetics is the use of insulin analogues, which are insulin-like molecules with altered pharmacokinetic parameters so that they are either absorbed more rapidly or slower compared to regular insulin after injection. A combinational treatment with these short- and long-acting insulin analogues provides the patient with normal blood glucose levels. These insulin analogues have been used for several decades, but recently some epidemiological studies found a correlation between the use of some of these compounds and cancer occurrence, especially breast cancer [2–5]. On the contrary, other epidemiological studies could not confirm these results and suggested that confounding factors (e.g. hyperinsulinemia and age of patients) might have caused this effect [6–11]. There are two main hypotheses by which insulin analogues might increase the risk of cancer [12]. First, the changes to the molecular structure of insulin affect the binding properties toward different receptors (e.g. the A isoform of insulin receptor (IRA) [13] or insulin-like growth factor 1 receptor (IGF1R) [14]). As a consequence these insulin analogues have an increased mitogenic potential. In this scenario the insulin analogues could act either as a tumor initiator by transforming benign or (pre)neoplastic cells, which often express increased levels of IRA and IGF1R [15], or as a tumor promoter by stimulating the increased growth potential of these cells. Second, insulin analogues might induce mutagenic action either directly or indirectly as a statistical consequence of the increased mitogenic potential. However, evidence for an indirect enhanced mutagenic effect due to insulin analogue treatment has never been observed and, therefore, the first hypothesis is the most plausible scenario. As indicated before, some insulin analogues have an increased binding potential toward the IGF1R [16] and/or a prolonged occupancy time for the IRA [17]. A simple evaluation of this effect has been the proliferative potential of insulin analogues, but the obtained results strongly depend on the used cell model and experimental procedures (reviewed in Bronsveld et al.) (Bronsveld, 2015 manuscript submitted) and are excluding the systematic evaluation of the actual role of the different insulin receptor families. We have developed a panel of MCF7 cell lines that express selectively either the IRA, the B isoform of insulin receptor (IRB) or IGF1R [18], which now allows us to differentiate the effect of individual insulin analogues on cellular signaling more precisely.

The downstream signaling of IRA and IGF1R is a complex diverse network leading to the activation of a diverse set of downstream cell signaling cascades and various transcription factors. The difference in activating either insulin receptor (INSR) or IGF1R signaling ultimately defines the cell biological outcome, roughly metabolic control versus promitogenic signaling respectively. The diversity of signaling events can be mapped using proteome-wide phosphoproteomics analysis [18]. Alternatively genome-wide transcriptomics may more broadly define the different signaling networks that are activated by either INSR or IGF1R. For the safety evaluation of novel chemical entities, transcriptomics-based profiling is often used to correctly classify the potential toxic properties [19, 20]. Given the differential activation of INSR and IGF1R by some insulin analogues, we anticipate that an IGF1R activation gene expression signature would be advantageous to define the mode of action of highly mitogenic insulin analogues. Therefore, in this study we used our MCF7 human breast cancer cell lines that differentially express the different insulin receptor family members [18]. We used transcriptomics to define gene sets involved in insulin analogue-induced mitogenic signaling. These genes are candidate mitogenic classifiers to predict the mitogenic potential of newly developed insulin analogues or growth factors in general that act on the IGF1R.

## Methods

### Primary cell isolation, cell line generation and cell culturing

Cell lines based on the human breast cancer MCF7 cell lines, which predominantly express the IRA, IRB or IGF1R have been described previously [18]. All MCF7 derivatives were cultured in RPMI 1640 medium (Gibco, Invitrogen, Carlsbad, CA, USA) supplemented with 10 % fetal bovine serum (FBS) and 100 U/mL penicillin-streptomycin (Invitrogen).

Primary human mammary cells have been isolated from cryopreserved biopsies of two individuals as described previously [21]. The two biopsies were obtained from two female patients who had undergone breast cancer-related surgery at the Leiden University Medical Center (LUMC). Procedures were followed according to the Dutch Medical Treatment Act and the study protocol was compliant with “the Code of proper secondary use of human tissue in the Netherlands” issued by the Dutch Federation of Medical Scientific Societies and approved by the Medical Ethics committee of the LUMC (P10.226). Specimens were coded anonymously in a way that they were not traceable back to the patient by laboratory workers. As much as possible fat tissue was removed from the human mammary biopsies, thereafter they were cut into 8-mm^3^ pieces, which were then dried and attached to the culture flask for 30 min. Twenty percent of FBS containing Dulbecco’s modified Eagle’s medium (DMEM)-F12 medium (Gibco/Invitrogen, Breda, the Netherlands) was gently added and refreshed every 5 days. Around the edges of the tissue, cells (mainly fibroblasts) started growing and after 3 weeks the culture flask was confluent with cells. The fraction of epithelial cells was enriched by multiple short trypsinization steps in which part of the fibroblasts were removed. For two more passages the cells were cultured in HuMEC Ready Medium (Gibco/Invitrogen). After this step the primary mammary cells were cultured in DMEM-F12 medium supplemented with 10 % FBS and 100 U/mL penicillin-streptomycin (Invitrogen).

### Insulin, insulin analogues and IGF1 in vitro stimulation

**Fig. 1:**
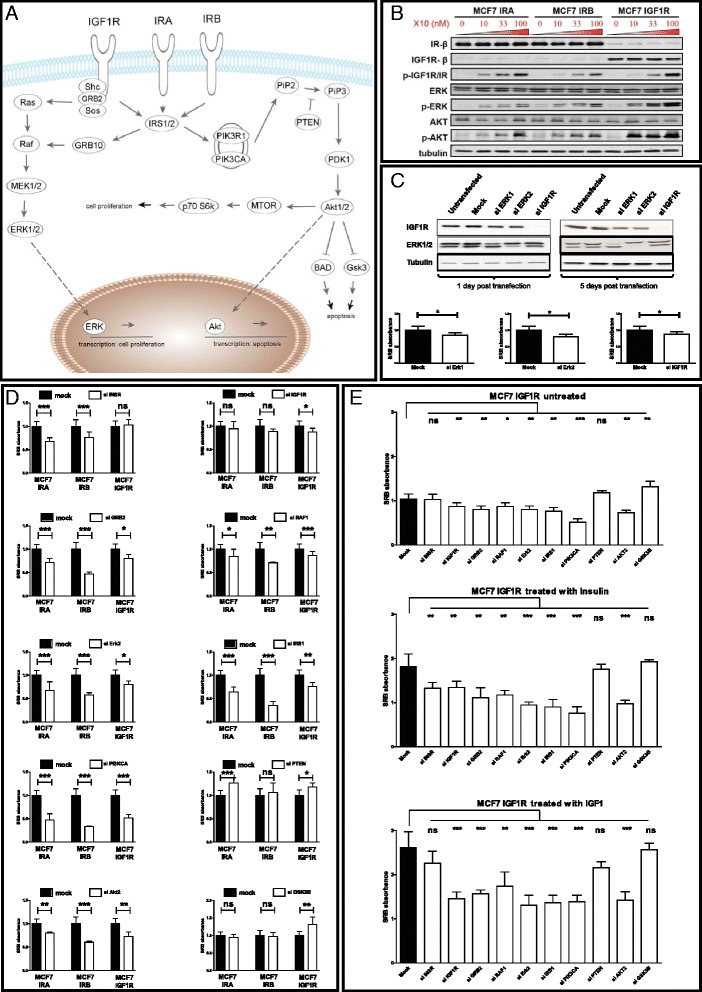
Knockdown of signaling components critical in the INSR and IGF1R pathway reveals common canonical core in IRA-, IRB- and IGF1R-induced proliferation signaling. **a** The canonical INSR and IGF1R signaling pathway with the focus on proliferative and apoptotic biological outcomes. **b** Western blot analysis of the cell line panel, based on the human breast cancer cell line MCF7 with stable retroviral overexpression (IRA, IRB and IGF1R) in combination with a stable short hairpin knockdown (INSR and IGF1R). Cells have been treated with 0, 10, 33 or 100 nM of insulin X10 for 30 min. Downstream signaling pathway activation of the receptors is intact as is indicated by the dose-dependent activation of p-ERK/p-AKT. **c** Western blot analysis of siRNA transfection efficiency in the MCF7 IGF1R cell line, 1 day and 5 days posttransfection and the effect of the knockdown on proliferation measured with the SRB proliferation assay. **d** The effect of transient knockdown of ten important signaling molecules (INSR, IGF1R, GRB2, RAF1, ERK2, IRS1, PIK3CA, PTEN, AKT2 and GSK3B) in the INSR and IGF1R signaling pathways on SRB proliferation measured in the different MCF7 derivatives (MCF7 IRA, MCF7 IRB and MCF7 IGF1R). **e** The effect of treatment and knockdown of key signaling molecules in INSR and IGF1R signaling on SRB proliferation measured in MCF7 IGF1R. (^*^ *p* <0.05, ^**^ *p* <0.01, ^***^ *p* <0.001). *IGF1R* insulin-like growth factor 1 receptor, *INSR* insulin receptor, *IRA* A isoform of INSR, *IRB* B isoform of INSR; *siRNA* small interfering RNA, *SRB* sulphorhodamine B

Prior to compound stimulation the cells were starved in 5 % charcoal/dextran-stripped fetal bovine serum (CDFBS, GE Healthcare HyClone, Utah, USA)-containing medium. Stimulations included: insulin neutral protamine Hagedorn (NPH) (Insuman Basal, Sanofi Aventis, Paris, France), insulin glargine (Lantus, Sanofi Aventis), first metabolite of glargine (M1, Sanofi Aventis), second metabolite of glargine (M2, Sanofi Aventis), glulisine (Apidra, Sanofi Aventis), lispro (Humalog, Eli Lilly, Indianapolis, IN, USA), insulin X10 (not marketed, Novo Nordisk, Bagsvaerd, Denmark), aspart (B28Asp, Novo Nordisk), detemir (Levemir, Novo Nordisk) and insulin-like growth factor 1 (IGF1) (Increlex, Ipsen, Basking Ridge, NJ, USA). All insulin analogues were dissolved in their original vehicle solutions [18]. For the in vitro experiments 1000× stock concentrations were prepared. Except for the first exposure experiment (Fig. 1c) in which a dose response of 10, 33 and 100 nM was used, all exposures were performed with a concentration of 10 nM.

### siRNA transfection

A transient transfection method with Smartpool siRNA mix (Dharmacon Technologies, Thermo Fisher Scientific, Lafayette, CO, USA) was used to test the effect of individual gene on cell proliferation. For this, 10,000 MCF7 cells per well were seeded in 96-well plates in complete growth medium. Twenty-four hours after seeding, 50 nM Smartpool siRNA mix was delivered to the cells using a standard transfection method with DharmaFECT 4 transfection reagent (Dharmacon Technologies) according to the company’s instructions. Twenty-four hours after transfection, the small interfering RNA (siRNA) transfection mixture was replaced with 5 % CDFBS starvation medium for drug treatment and sulphorhodamine B (SRB) proliferation assay.

### Sulforhodamine B colorimetric assay determining cell proliferation

A SRB assay was used to measure the total amount of protein as a measure for cell proliferation. Transfected and drug-treated cells in 96-well plates were fixed with 30 μl 50 % trichloroacetic acid directly added to 100 μl of assay medium per well for 1 h at 4 °C on a shaker, washed five times with distilled water and air-dried. Fixed cells were stained with 60 μl of 0.4 % SRB (dissolved in 1 % acetic acid) at room temperature on a shaker for 30 min. After the SRB protein binding, the plates were washed five times with 1 % acetic acid to remove unbound dye and air-dried between the washing steps. Next, the protein-bound SRB in each well was solubilized in 200 μl 10 mM unbuffered Tris solution (pH >10) for 10 min on a plate shaker. Absorbance was measured at 530 nm with a FLUOstar OPTIMA plate reader (BMG Labtech, Offenburg, Germany).

### Western blotting

Western blotting was used to determine the knockdown efficiency of the siRNA transfection. To prepare cell lysates for Western blot analysis, cells were washed two times with ice-cold phosphate-buffered saline (PBS) and lysed with 1 × SPB with 1:20 β-mercaptoethanol. Samples were boiled at 95 °C for 5 min and stored at −20 °C. Before loading, samples were denatured at 95 °C for 5 min. A total of 20 μl (about 30 ug) protein solution per lane was separated by SDS-polyacrlyamide gel electrophoresis on a 7.5 % acrylamide gel and electrophoretically transferred to a polyvinylidene fluoride membrane (Merck Millipore, Billerica, MA, USA). Prior to primary antibody probe, membranes were blocked for 1 h at room temperature with 5 % bovine serum albumin (BSA) or 5 % milk in Tris-buffered saline and Tween 20 (TBST) buffer (100 mM Tris, pH 7.4, 500 mM NaCl, 0.05 % Tween 20). ERK, AKT, PTEN and tubulin antibodies were probed in 1 % BSA-TBST buffer, whereas IGF1Rβ antibodies were probed in 1 % milk-TBST buffer. Horseradish peroxidase (HRP)-conjugated secondary antibody incubation was performed in 1 % BSA-TBST or 1 % milk-TBST buffer, corresponding to the primary antibodies used. Protein bands were visualized by using the ECL (Amersham) method, after which the membrane was scanned by using a Typhoon 9400 imager (GE Healthcare, Amersham, UK). Anti-phospho-Akt (Ser473) and anti-phospho-Erk (Thr202, Tyr204) have been purchased from Cell Signaling Technology, Danvers, MA, USA). For a detailed description of the methods and origin of the antibodies we refer to our prior publications [18, 22].

### Microarray studies

For the microarray, the cells were seeded at a confluence of 60 % in 6-cm plates, starved for 2 days in 5 % CDFBS-containing medium, followed by 1 h or 6 h compound stimulation (10 nM) in serum-free medium. Small and large RNA was isolated and purified using NucleoSpin® miRNA isolation kit (Macherey-Nagel, Düren, Germany) according to the manufacturer’s instructions. RNA quality and integrity were assessed by using the Agilent 2100 Bioanalyzer System (Agilent Technologies, Santa Clara, CA, USA). The Affymetrix 3′ IVT Express Kit (Affymetrix, Santa Clara, CA, USA) was used to synthesize biotin-labeled cRNA, and this was hybridized to an Affymetrix HG-U133 Plus PM Array plate reader. Probe annotation was performed using the hgu133plus2.db package and probe mapping was performed with the hgu133plus2cdf package installed using Bioconductor version 3.0. Probe-wise background correction (Robust Multi-array Average expression measure), between-array normalization (quantile normalization) and probe set summaries (median polish algorithm) were calculated with the RMA function of the Affymetrix package (Affy package version 1.38.1) [23]. The normalized data were statistically analyzed for differential gene expression using a linear model with coefficients for each experimental group [24]. A contrast analysis was applied to compare each exposure with the corresponding vehicle control. For hypothesis testing the moderated t-statistic by empirical Bayes moderation was used followed by an implementation of the multiple testing correction of Benjamini and Hochberg [25] using the LIMMA package [26]. The microarray data is publically available at the Gene Expression Omnibus (GEO) database via accession number GSE65398.

### RT-qPCR

For the qPCR analysis, messenger RNA from MCF7 cells (80 % confluent 6-well) or mammary glands (30 μg tissue) was isolated/purified using NucleoSpin® miRNA isolation kit (Macherey-Nagel). cDNA was made using the universal cDNA synthesis kit (Exiqon, Vedbaek, Denmark). qPCR was performed in triplicate using SYBR Green PCR (Applied Biosystems, Carlsbad, CA, USA) on a 7900HT Fast Real-Time PCR system (Applied Biosystems). Primers targeting the mitogenic classifiers have been manually designed and are listed in the additional material (Table S1 in Additional file 1). qPCR data were collected and analyzed using SDS2.3 software (Applied Biosystems). Relative gene expression was calculated after correction for β-actin expression using the 2^-ΔΔQ^ method. Data are presented as fold change (or log2 fold change) compared to vehicle stimulation.

### Animal experiments

Forty female 12-week-old inbred FVB/NRj mice were obtained from Janvier Labs, Orleans, France. Housing and experiments were performed according to the Dutch guidelines for the care and use of laboratory animals (UL-DEC-14020). RM2 food SDS (Technilab-BMI, Someren, Holland) and water were provided ad libitum. Animals received a single subcutaneous injection of 100 μl compound/vehicle solution. The doses were chosen so that the glucose drop was constant among the different compounds (see Figure S3A in Additional file 2) (glargine and insulin 100 nmol/kg, X10 1200 nmol/kg and IGF1 12.5 mg/kg). One or six hours after the injection the mice were sacrificed, blood was collected (mini collect, Greiner/Omnilabo, Breda, Holland), blood glucose levels were measured (Freestyle light, 70812–70, Abbott Laboratories, Abbott Park, IL, USA), the third and fourth mammary glands were isolated and used for Western blot protein quantification and quantitative PCR respectively [18, 22]. For each condition (treatment/time point) four mice were included.

### Statistical analysis

For the statistical analysis of the microarray data, R (version 3.1) software was used. The rest of the analysis was performed with Graphpad Prism version 4.00 (Graphpad Software, San Diego, CA, USA). Student’s *t* tests were used to determine significance between conditions. *P* values lower than 0.05 were considered to be significant. In all graphs the error bars represent standard deviations.

## Results

### Mitogenic signaling is regulated via highly similar signaling cascades in the INSR and IGF1R signaling pathway

To better understand the involvement of the IRA, IRB and IGF1R pathways (Fig. 1a) in the context of mitogenic signaling of insulin analogues, we used our previously described human MCF7 breast cancer cell lines that express either IRA, IRB or IGF1R [18]. Exposure of these individual cells to the promitogenic insulin analogue X10 that activates both the INSR and the IGF1R, resulted in intact downstream signaling cascades in all three cell lines, indicating functionality of the receptors (Fig. 1b). As a next step, we wanted to ensure that the IRA, IRB and IGF1R are not entirely different regarding their key intracellular proliferative signaling pathways. For this purpose, we tested the proliferative potential of the cells after knockdown of several key signaling molecules in these pathways (Fig. 1a). As a first step, we optimized the knockdown efficiency using IGF1R and ERK1/2 as controls. The knockdown efficiency of IGF1R was almost 100 % and constant over 5 days of culturing; the knockdown efficiency of ERK1 and ERK2 was around 50 % after day 1 up to 95 % at day 5 (Fig. 1c), we assume that the knockdown efficiency is constant for other targets but obviously it was practically not feasible to test them all in this study. To assess the proliferative and antiapoptotic effects of these knockdowns, we used the SRB proliferation assay. After knockdown of ERK1/2 and IGF1R, the amount of cells after 5 days of culturing was significantly decreased with 25 % (Fig. 1c), indicating that the SRB proliferation assay is a sensitive assay to pick up any antiproliferative effects.

Next, we determined the effect on proliferation of ten individual signaling molecules that are key in the INSR/IGF1R signaling pathway (INSR, IGF1R, GRB2, RAF1, ERK2, IRS1, PI3KCA, PTEN, AKT2, GSK3B) in untreated IRA, IRB and IGF1R MCF7 cell lines (Fig. 1d). As expected, the INSR knockdown only significantly affected the proliferative behavior in the IRA and IRB cell line. Similarly, the siIGF1R only significantly reduced the proliferative behavior in the MCF7 IGF1R cell line. Transient knockdown of downstream targets in the MAPK signaling cascade (GRB2, RAF1 and ERK2) all had a significant inhibiting effect on proliferation. Also knockdown of targets in the PI3K signaling cascade (IRS1, PI3KCA and AKT2) had a significantly reduced proliferative effect in all cell lines. It has to be noted that different members of the AKT family have redundant functions and can therefore take over the loss of function of the silenced member. We expect that a knockdown of all three members of AKT would lead to an even stronger effect on cell proliferation [27]. As anticipated, a knockdown of PTEN increased the proliferative potential in these cells, since PTEN acts as a tumor suppressor through dephosphorylation of phosphoinositide-3 phosphate, thereby negatively regulating PI3K signaling. Also GSK3B knockdown showed an increase in proliferation. The antiapoptotic effects of GSK3B are likely to cause this effect, which was only detected in the IGF1R-overexpressing cell line. Finally, the proliferative potential of nonstimulated MCF7 IGF1R cells treated with different siRNAs was compared to insulin and IGF1 treatment conditions (Fig. 1e). The effect of the stimulation itself was clearly detectable as the SRB absorbance increased from 1.04 (untreated, upper graph) to 1.81 (insulin treated, middle graph) to 2.61 (IGF1 treated, lower graph) in the mock condition. Furthermore the effects of the different siRNA knockdowns on proliferation became more prominent in the stimulation conditions. Interestingly, in the insulin-stimulated condition the siINSR significantly affected proliferation in the IGF1R cell line, suggesting that the low levels of INSR in this cell line (Fig. 1b) are involved in proliferation once stimulated with high levels (10 nM) of insulin.

It could be argued that the effects described above are not (solely) due to promitogenic effects, since the INSR/IGF1R signaling pathway can also induce antiapoptotic effects (see Fig. 1a). These antiapoptotic effects could also lead to more cells and thus a higher SRB assay readout. To investigate this, we determined the apoptotic fraction with a FACS analysis upon stimulation with the different growth factors (insulin, glargine, X10, IGF1) at 0, 10 and 100 nM. As expected, we found a slightly, but dose-dependent, higher fraction of apoptotic cells in the growth factor-stimulated cells (approximately 6 %) versus the unstimulated (approximately 4 %) (data not shown). Since this is such a small difference, we assume that the antiapoptotic effects play a minor role compared to the promitogenic effects in the growth factor stimulation experiments.

In conclusion, these combined data indicates that the core signaling pathways involved in cell proliferation are similar in the entire MCF7 INSR family cell panel, allowing us to use this panel to further unravel the signaling events that can differentiate between INSR versus IGF1R acting insulin analogues.

### Insulin analogues trigger different transcriptomes in the different cell lines

**Fig. 2:**
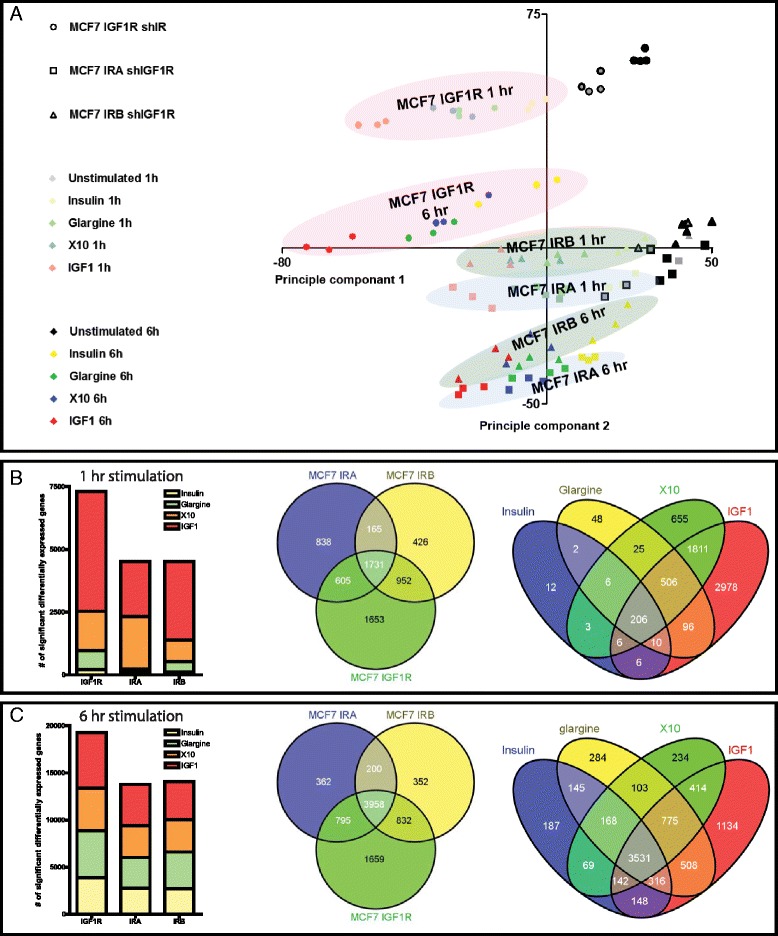
Experimental setup of microarray experiment. **a** Two-dimensional principal component analysis plot of the microarray gene expression data. A clear separation of the different treatments (indicated by the different colors of the dots), cell lines (different shapes) and time points (light; 1 h vs. dark; 6 h) could be observed. The triplicates or quadruplicates are as expected very close to each other. **b** Number of significantly differentially expressed genes between the different cell lines at T = 1 h. The first Venn diagram shows overlap of the significant differentially expressed genes (DEGs) per cell line at T = 1 h. The second Venn diagram shows overlap of the significant DEGs per treatment at T = 1. **c** As in (**b**) but then for T = 6 h

To detect the differences in gene expression levels between the different cell lines, we next performed a microarray experiment using the same cell line panel (Fig. 1b). We included five different stimulation conditions (vehicle, insulin, glargine, X10 and IGF1 stimulation). This allowed the comparison of the transcriptomes of the different treatments. We also included two different time points, thus enabling observation of possible time dynamics. Using a principal component analysis (PCA) a clear separation of the different treatments and cell lines conditions was observed (Fig. 2a). The PCA indicated that the transcriptome after IGF1 treatment was most different from vehicle stimulation. Glargine and X10 treatments cluster together and insulin treatment is closest to the vehicle-treated situation. Triplicate (or quadruplicate for the vehicle) samples from independent biological experiments were close to each other, indicating a strong robustness of the assay.

We determined the significantly differentially expressed genes (DEGs) per condition (1 h stimulation, Fig. 2b; 6 h stimulation, Fig. 2c). Most DEGs were observed in the IGF1R cell line, which is consistent with the strongest separation of this cell line in the PCA analysis (Fig. 2a). We combined all DEGs per cell line and determined the overlap from the different treatments. There was a 43 % overlap in the DEGs between the different cell lines. We also compared the treatment-specific responses independent of the cell line (right Venn diagram Fig. 2b/c). Again, IGF1 treatment has the biggest impact on the transcriptome, and the highest overlap with the X10 and glargine treatment. The total number of DEGs 6 h after stimulation (Fig. 2c) is considerably larger compared to the 1 h stimulation (Fig. 2b). After 6 h of stimulation there was a large overlap of DEGs among the different cell lines as well as treatments (3531), suggesting that at this time point more general mechanisms were activated that are similar for the different treatment conditions. Venn diagrams of all the different conditions are presented is the additional material (Figure S1 in Additional file 3). A noteworthy finding is the very high number of DEGs in the X10 stimulation via the IRA at T = 1 h.

### Differential pathway activation by the various insulin analogues

**Fig. 3:**
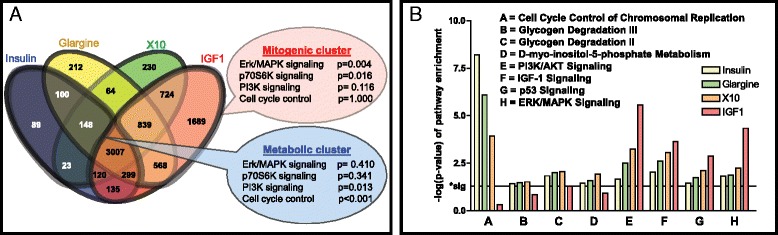
Pathway enrichment analysis of differentially expressed gene lists. **a** Separate gene clusters were defined based on the Venn diagram of MCF7 IGF1R. The mitogenic gene cluster consists of all DEGs in IGF1 treatment alone and the combinations of IGF1 with glargine and/or X10 treatment. Similarly, a metabolic gene cluster was defined including the insulin-specific DEGs with combinations of the other insulin analogues. An Ingenuity Pathway Analysis (IPA) was performed that revealed an enrichment of the ERK/MAPK and p70S6K signaling pathways in the mitogenic cluster whereas the PI3K and cell cycle control signaling pathways were enriched in the metabolic cluster. **b** An IPA analysis was performed on the DEGs of individual treatments including all cell lines and the different time points. As expected the metabolic signaling (A t/m D) was upregulated after stimulation with metabolic compounds (insulin, glargine and X10). IGF1 stimulation led to a very significant upregulation of PI3K/AKT, ERK/MAPK, IGF1 and p53 signaling. For insulin signaling these pathways were also enriched but less significantly. *DEGs* differentially expressed genes; *IGF1R* insulin-like growth factor 1 receptor

To further understand the biological pathways upregulated by these different compounds we performed an Ingenuity Pathway Analysis (IPA), focusing on the MCF7 IGF1R cell line using both time points (Venn diagram Fig. 3a). A *mitogenic cluster* was defined that included all DEGs of IGF1 treatment only, or IGF treatment in combination with X10 and/or glargine treatment. We included glargine treatment in this cluster as glargine, like X10, is highly mitogenic in the absence of serum [18]. In a similar way a *metabolic cluster* was defined, including all DEGs of insulin treatment only, or insulin treatment in combination with glargine and/or X10 since all these compounds are known to have a strong metabolic effect in vivo. Using IPA we found ‘ERK/MAPK’ and ‘p70S6K’ signaling pathways significantly enriched in the *mitogenic cluster*, while the ‘PI3K’ and ‘cell cycle control’ pathways were not enriched. For the *metabolic cluster* the IPA results were the other way around. We also performed IPA analysis on the individual treatment DEG lists. ‘Cell cycle control of chromosomal replication’ was highly enriched after treatment with compounds with a high affinity for the INSR (insulin, glargine and X10). Other metabolic processes like glycogen degradation and D-myo-inositol-5-phosphate metabolism were also enriched in the DEGs of these insulin molecules. On the other hand PI3K/AKT, IGF1, p53 and ERK/MAPK signaling were more enriched for the insulin-like molecules that also have a strong affinity for the IGR1R.

### A classifier gene set predictive for the promitogen action of insulin analogues

**Fig. 4:**
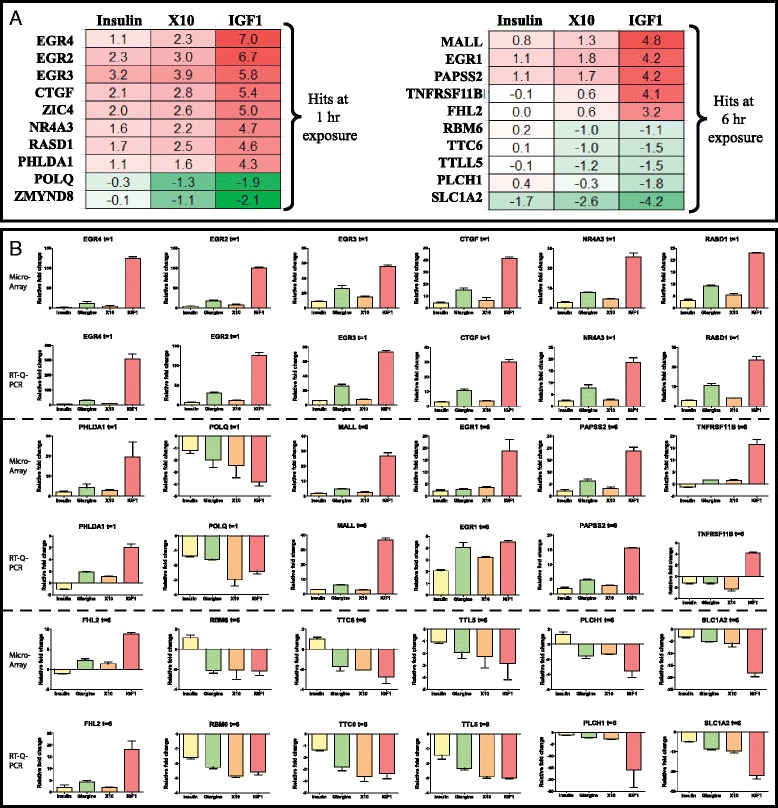
Twenty mitogenic classifier genes discriminating between insulin and X10/IGF1 signaling. **a** Twenty genes (ten for each time point) were defined of which the gene expression was most significantly up- or downregulated in X10/IGF1 vs. insulin in the MCF7 IGF1R cell line (based on a variance test), values are presented as log2 fold changes. **b** Validation of 18 of these mitogenic classifier genes was successful with RT-qPCR. A comparison is given of the microarray (*top panel*) vs. RT-qPCR (*lower panel*) gene expression of the mitogenic classifiers. Expression is indicated as fold changes relative to unstimulated MCF7 IGF1R. *IGF1* insulin-like growth factor 1, *IGF1R* IGF1receptor

To evaluate which genes drive the strong mitogenic responses of IGF1R signaling we performed a variance test with selected genes showing a strong up- or downregulation after strong activation of the IGF1R. For this we selected IGF1 and X10 exposures and contrasted this with the weak mitogenic response inducer insulin. We excluded glargine for the selection. In total we selected the top ten hits in both the 1 h and 6 h hit lists (Fig. 4a). Interestingly, many of these genes are known to play a role in mitogenic processes, including the early growth response (EGR) genes (all four EGR genes are in the top 20 gene list). Most of these genes have not directly been linked to the INSR or IGF1R signaling pathway so far. Next we validated these candidate genes using RT-qPCR with a separate independent set of samples. For 18 of these mitogenic classifier genes the RT-qPCR validation was successful and showed a highly similar trend for insulin, X10 and IGF1 conditions (Fig. 4b). For ZIC4 and ZMYND8 the expression was probably too low since no amplicon was detected even after 40 cycles. Finally, we evaluated the effect of glargine on the expression of these classifier genes. Importantly, the overall expression of the classifier genes after glargine treatment was more similar to X10 than to insulin treatment.

### Validation of mitogenic classifiers through testing of commercially available insulin analogues

**Fig. 5:**
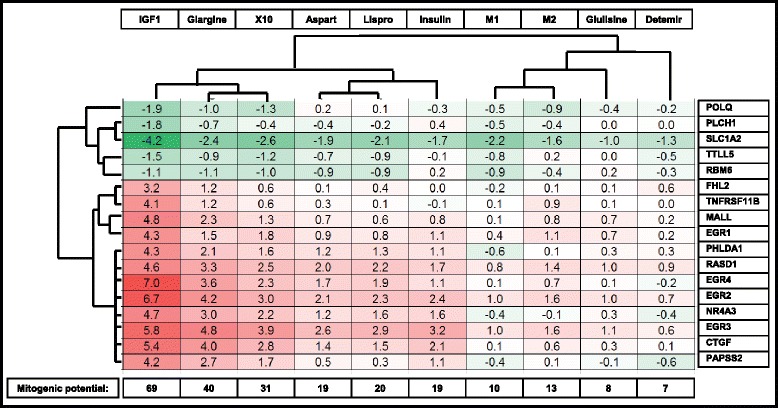
The relative mitogenic potential of various insulin analogues determined by the classifier gene expression. MCF7-IGF1R cells were exposed to IGF1, X10, glargine, insulin, aspart, lispro, M1, glulisine, M2 and detemir and the gene expression of the mitogenic classifier genes was measured with RT-qPCR. A hierarchical clustering of the log2 fold changes (compared to vehicle stimulation) is shown. The mitogenic potential score is the absolute sum of the absolute values of log2 fold changes of the mitogenic classifiers. *IGF1* insulin-like growth factor 1, *IGF1R* IGF1receptor

We hypothesized that the expression of the mitogenic classifier genes could predict the mitogenic outcome of other insulin analogues. We performed an exposure experiment with MCF7 IGF1R cells including all commercially available insulin analogues (glargine, aspart, lispro, glulisine, determir). Since glargine showed expression of the predictive genes (Fig. 4b), and since glargine is rapidly metabolized into two metabolites (M1 and M2) in serum, we also included M1 and M2 in our study. A hierarchical clustering of the expression of all the tested classifier genes after stimulation with the different insulin analogues was performed (Fig. 5). This resulted in the clustering of glargine with IGF1 and X10, while the glargine metabolites M1 and M2 clustered with other relatively nonmitogenic insulin analogues. We calculated a ‘relative mitogenic potential’, which was determined as the sum of the absolute values of log2 fold changes of the expression of mitogenic classifiers of one compound treatment. As expected the ‘relative mitogenic potential’ was highest for IGF1 (69), followed by glargine (40) and X10 (31). The mitogenic potential of insulin (19) was very similar to that of aspart (19) and lispro (20). M1 (10), M2 (13), glulisine (8), and determir (7) showed a lower predicted mitogenic potential compared to regular insulin.

### Validation of mitogenic classifier genes in vitro in primary human mammary gland cells and in vivo in mouse mammary glands

**Fig. 6:**
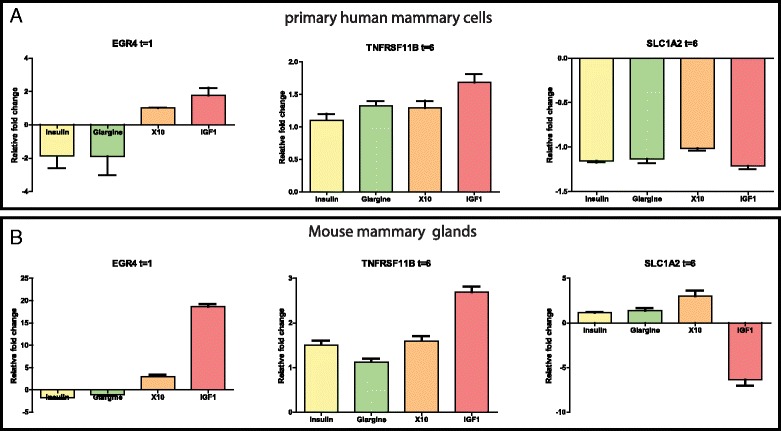
Validation of insulin analogue mitogenic classifier genes in primary human mammary cells and in vivo mouse mammary glands. **a** Primary cells isolated from two different human mammary biopsies were exposed to insulin, glargine, X10 or IGF1 and the gene expression of EGR4, TNFRSF11B and SLC1A2 were determined by RT-qPCR. Values are presented as fold changes relative to vehicle-treated cells. **b** Wild-type FVB mice (four per condition) were subcutaneously injected with the above mentioned compounds, RNA from the mammary glands was isolated and the gene expression of EGR4, TNFRSF11B and SLC1A2 was measured by RT-qPCR and indicated as fold change relative to gene expression of vehicle-injected mice. *IGF1* insulin-like growth factor 1

To further validate the insulin analogue mitogenic classifier genes we tested additional in vitro and in vivo models. We first determined the robustness of the insulin analogue mitogenic classifier genes in primary cultured cells isolated from human mammary glands. These cells were anticipated to be the main target for increased mitogenic signaling of insulin analogues in diabetic patients. Primary cells were isolated from two independent individuals and exposed to the different insulin-like molecules. The activation of the INSR/IGF1R pathway was validated by Western blotting (Figure S2 in Additional file 4) and a clear activation of the INSR/IGF1R as well as the PI3K/AKT signaling pathway was observed. Next, the gene expression levels of three mitogenic classifier genes that were upregulated (EGR4 and TNFRSF11B) or downregulated (SLC1A2) in MCF7 IGF1R cells were measured (Fig. 6a). Although the fold change expression of these three genes in these primary human mammary cells was not as profound compared to the MCF7 IGF1R cells, in general the same direction of expression was observed.

In addition, we investigated these three classifier genes in vivo in the mammary glands of mice treated with the different insulin analogues. In this experiment 40 wild-type FVB mice received a subcutaneous injection of vehicle, insulin, glargine, X10 or IGF1. A very clear and constant drop in the glucose levels was observed 1 h after the injections of insulin, glargine, X10 and IGF1, indicating that these compounds did induce the expected pharmacological response (see Figure S3A in Additional file 2). The glucose levels returned to their normal levels (5 mmol/L) 6 h after the injection. We then investigated the activation of the INSR and IGF1R (see Figure S3B and C in Additional file 2). One hour after the insulin analogue injections a clear upregulation of p-AKT was observed, while after 6 h the p-p70S6K levels were significantly (*p* = 0.0022) upregulated. Also the insulin analogue mitogenic classifier genes showed a very clear modulation after treatment (Fig. 6b). Thus, EGR4 was even induced up to 18 times after IGF1 treatment and X10 also showed a clear upregulation of this candidate gene compared to no stimulation. Similarly, after 6 h treatment IGF1 induced TNFRSF11B and downregulated SLC1A2 levels. For these latter gene changes none of the other insulin-like molecules caused a significant change in gene expression. Gene expression in glargine conditions showed a similar trend as regular insulin, suggesting that glargine is rapidly metabolized into M1 and M2 in vivo, which are known to be compounds with a low promitogenic signaling potential [18].

## Discussion

It is well established that insulin has strong metabolic effects and in addition mild promitogenic characteristics [14]. Small changes in the structure of insulin have improved the pharmacokinetic parameters so that the use of the insulin analogue is more convenient for diabetic patients. Yet, these small structural changes might also increase the binding affinity of insulin analogues towards the IGF1R and, consequently, increase the mitogenic potency of these molecules compared to regular insulin. Current in vitro systems that are used to determine mitogenic potential of insulin analogues are largely based on the proliferation capacity and do not take into account the molecular mechanisms of receptor signaling (Bronsveld 2015 manuscript submitted). In this study we used a transcriptomics approach to assess the preferential activation of promitogenic signaling pathways by insulin analogues. We identified a subset of classifier genes that can be used to define the primary mode of action of insulin analogues. Moreover, we demonstrated that these classifier genes can be translated to primary human mammary cells as well as mouse mammary glands in vivo. These mechanism-based novel predictive genes are likely a more reliable method to classify the proliferative potency of insulin analogues that act preferably on the IGF1R.

For the safety profiling of insulin analogues this increased mitogenic potential is critical. Currently, there is still a debate on the mechanism of such an increased mitogenic potential: on one hand the high binding affinity toward the IGF1R, while on the other hand a prolonged occupancy time toward the IRA is suggested causative [17]. In our current study, we have been in the unique situation to evaluate these mechanisms in our MCF7 cell line panel. We found 40 % more differentially expressed genes in the MCF7 IGF1R cell line after X10 and glargine stimulation compared to the MCF7 IRA cell line. These results suggest that the IGF1R is the main receptor that is mediating downstream promitogenic signaling after insulin analogue stimulation. This is in line with our previous study in which we tested the proliferative potency of nine insulin-like molecules using the same MCF7 cell line panel and found that X10 and glargine induce proliferation more profoundly in the MCF7 IGF1R cells than in the MCF7 IRA cells [18]. For this reason we based the further mitogenic classifier analysis on the MCF7 IGF1R cell line.

For the mitogenic classifier hit selection, a training set was based on microarray gene expression of three compounds, in which insulin served as the reference compound with a low mitogenic potency. X10 and IGF1 served as two insulin-like molecules with a strong mitogenic potential. This resulted in a total of 20 genes either up- or downregulated at 1 or 6 h after IGF1 and X10 treatment that most strongly differed from the insulin effect. Many of these genes have been associated with mitogenic signaling but so far not directly linked to INSR/IGF1R signaling. Strikingly, various early growth response genes were identified: EGR1 [28], EGR2 [29], EGR3 [30], EGR4 [31] which are all well known to promote proliferation, survival and/or invasion pathways. MALL, FHL2 [32], PHLDA [33], NR4A3 [34] and CTGF [35] are known oncogenic factors and its gene expression is negatively associated with tumor-free survival and/or proliferation. Interestingly, genes (POLQ and RBM6) that were downregulated after X10/IGF1 stimulation have been linked to proapoptotic and antiproliferative effects [36, 37].

Glargine is the most frequently prescribed antidiabetic insulin analogue. There are conflicting conclusions regarding the intrinsic mitogenic potential of insulin glargine [38, 39]. We purposely excluded our glargine transcriptome analysis from the training set to identify candidate predictive classifier genes for promitogenic signaling by insulin analogues. This allowed us to unravel the potency of glargine as a promitogenic insulin analogue. Interestingly, the mitogenic potential of glargine was even higher compared to insulin X10 (Fig. 5). This is in full agreement with the kinase activation measurement of INSR/IGF1R pathway components in our previous study [18]. In diabetic patients glargine is rapidly processed by enzymes in the serum into two metabolically active compounds, M1 and M2, in which M1 is most prominent metabolite [40]. Therefore, we also determined the mitogenic potential of M1 and M2 based on the gene expression profiles of the mitogenic classifier genes and we observed that both metabolites have a mitogenic score that is even lower than insulin. This is in agreement with previous studies in which the mitogenic potential was based on IGF1R binding affinity, kinase activation or proliferation assays [16, 18].

**Fig. 7:**
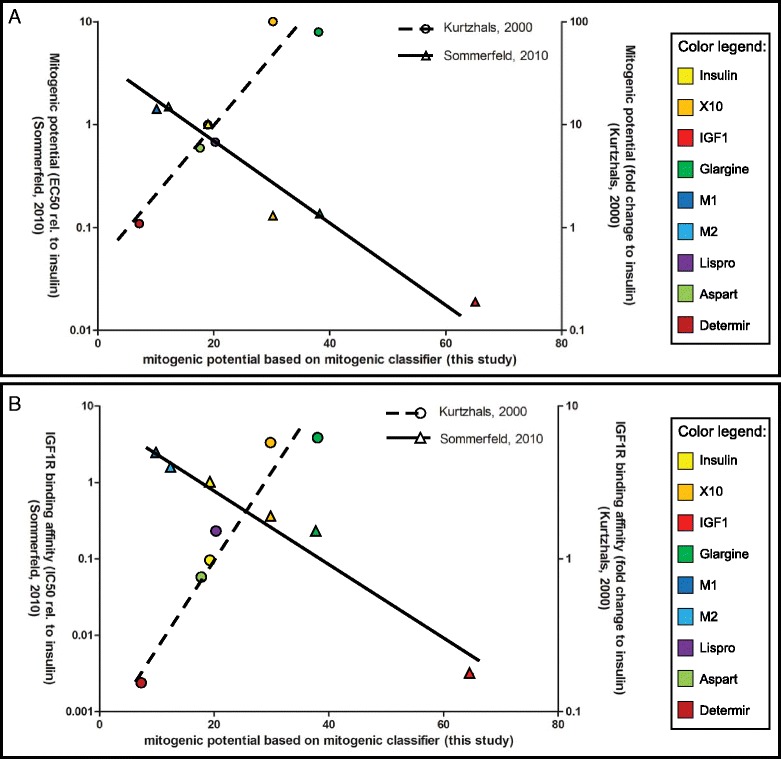
Correlation between mitogenic classifier score and the enhanced proliferation or IGF1R binding. **a** A correlation is presented of the mitogenic potential based on the mitogenic classifier analysis (*x-axis*) vs. the mitogenic potential as determined by other studies with proliferation assays. Sommerfeld et al. [16] expressed this score as EC50 value from their proliferation curves (*left y-axis*). Data from Kurtzhals et al. [41] are expressed as fold changes compared to insulin (*right y-axis*). **b** A similar correlation graph with the mitogenic potential from this study plotted against the IGF1R binding affinity according to two other studies. Sommerfeld et al. [16] expressed this score as IC50 value from their competition assay curves (*left y-axis*). Data from Kurtzhals et al. [41] are expressed as fold changes compared to the binding affinity of insulin toward the IGF1R (*right y-axis*) (data adapted from Kurtzhals et al. [41] and Sommerfeld et al. [16]). *IGF1R* insulin-like growth factor 1 receptor

Two other studies also performed a mitogenic assessment of a panel of insulin analogues. These studies included a proliferation assay (Kurtzhals et al. [41]) and an IGF1R affinity evaluation [16]. We systematically compared our mitogenic classifier gene score with these two independent functional readouts (Fig. 7). There was a striking correlation between our classifier scoring (based on Fig. 5) and both the proliferation and IGF1R affinity. These combined datasets demonstrate that IGF1, X10 and glargine have a higher mitogenic potential compared to insulin, which is associated with a high affinity for IGF1R. Aspart and lispro have a mitogenic potency similar to each other. Determir and the two metabolites of glargine (M1 and M2) have a lower mitogenic index compared to regular insulin, associated with a low affinity for the IGF1R.

Some epidemiological studies suggest a correlation between insulin glargine use and breast cancer occurrence in the diabetic patients [4]. Since glargine might promote proliferation of mammary epithelial cells in vivo, we wanted to test whether expression of some of our classifier genes could be translated from MCF7 cells to primary human mammary cells. We could confirm a similar gene expression trend in primary human mammary cells after stimulation with insulin, glargine, X10 and IGF1 as in MCF7. Yet, the gene expression fold changes in the primary human mammary cells were far lower compared to the MCF7 IGF1R cells. The reason for this can be partly due to the isolation procedure, which did not result in a pure population of mammary epithelial cells. Overall, the effect of glargine in the primary human mammary cells was not as profound as for IGF1 and X10.

Previously, we demonstrated that IGF1 and X10 significantly promote tumorigenesis in a conditional mammary gland tumor mouse model [22]. Glargine did not significantly enhance this tumorigenesis. Therefore, we also evaluated the translation of our classifier genes to the in vivo situation and determined the gene expression changes in the mammary glands of mice that received a subcutaneous injection of the insulin-like molecules insulin, IGF1, X10 and glargine. We could validate the in vitro effect of IGF1 in the in vivo situation, indicating that a true IGF1R response can be observed in this model. X10 showed some correlation with the effect of IGF1. Yet, in contrast to the in vitro data, the gene expression profiles of glargine were more similar to insulin than to X10/IGF1. This effect is very likely caused by the metabolism of insulin glargine by factors in the serum of the blood of the mice (similar to the glargine conversion in human serum). We therefore speculate that the observed promitogenic signaling events of glargine in our in vitro breast cancer cell line models are presumably not occurring under in vivo conditions in the mammary gland. Yet, we cannot exclude that IGF1R-mediated responses by glargine take place in other tissues in vivo.

## Conclusions

In the current study we propose a new robust classifier gene set that allows the quick, robust and quantitative analysis of the promitogenic potential of newly developed insulin analogues. These classifiers can be used within the pharmaceutical industry as well as in a regulatory setting to define the safety profile of insulin analogues as well as other growth factors that might act on the IGF1R.

## Additional files

Additional file 1: Table S1. Sequences of used primers. All the primers used for the RT-qPCR experiments including primers targeting human and mouse genes. Additional file 2: Figure S3. Mammary gland protein levels of 40 FVB mice subcutaneously injected with insulin-like compounds. (*A*) The blood glucose levels (mmol/L) have been measured 1 h and 6 h after subcutaneous injections with different insulin-like molecules. (*B*) Western blotting of the receptors and kinases in the INSR and IGF1R pathway measured in the mammary gland tissue; samples were from 1 h (*left graph*) or 6 h (*right graph*) after the subcutaneous injections of the presented insulin-like molecules. (*C*) the quantification of activated kinases (Erk, Akt, p70S6K, FOXO1/O3) relative to the endogous control (EC), a sample that was loaded on every blot. N = 4. Additional file 3: Figure S1. Venn diagrams of the differentially expressed genes per cell line, time point and treatment showing overlap of genes between the different conditions. (*A*) Venn diagram of the early regulators (t = 1 h). The *blue circle* represents the hits in the IRA cell line, *yellow* the IRB cell line and *green* the IGF1R cell line. The first graph shows the number of hits after insulin treatment, the second graph glargine, the third X10 and the last graph shows the number of hits after IGF1 treatment. (*B*) The Venn diagrams of the late regulators (t = 6 h). Additional file 4: Figure S2. Protein levels of primary human mammary cells stimulated with insulin-like compounds. (*A*) Primary human mammary gland cells were treated with different insulin-like molecules followed by Western blotting for various INSR and IGF1R signaling pathway components. (*B*) Quantification of Western blot data of the p-IGF1R/p-IR, p-Akt and p-Erk. The y-axis represents the average protein expression level compared to vehicle exposure.

## Abbreviations

BSA: bovine serum albumin
CDFBS: charcoal/dextran-stripped FBS
DEG: differentially expressed genes
DMEM: Dulbecco’s modified Eagle’s medium
EGR: early growth response
FBS: fetal bovine serum
GEO: Gene Expression Omnibus
HRP: horseradish peroxidase
IGF1: insulin-like growth factor 1
IGF1R: IGF1 receptor
INSR: insulin receptor
IPA: Ingenuity Pathway Analysis
IRA: A isoform of INSR
IRB: B isoform of INSR
M1: first metabolite of glargine
M2: second metabolite of glargine
NPH: neutral protamine Hagedorn
PBS: phosphate-buffered saline
PCA: principal component analysis
siRNA: small interfering RNA
SRB: sulphorhodamine B
TBST: Tris-buffered saline and Tween 20
