# Supplementary material for: Alternative signaling network activation through different insulin receptor family members caused by pro-mitogenic antidiabetic insulin analogues in human mammary epithelial cells
Source: Breast Cancer Res. 2015 Jul 19;17(1):97. doi: 10.1186/s13058-015-0600-5 (PMC4506606; doi:10.1186/s13058-015-0600-5)
Supplement: Additional file 1: Table S1. — Sequences of used primers. All the primers used for the RT-qPCR experiments including primers targeting human and mouse genes. [file 13058_2015_600_MOESM1_ESM.docx]

**Additional Table 1. Sequences of primers.** All the primers used for the RT-Q-PCR experiments including primers targeting human and mouse genes.

| **Target gene** | **Orientation** | **species** | **Sequence 5' to 3'** |
| --- | --- | --- | --- |
| egr1 | forward | mouse | cctatgagcacctgaccaca |
|  | reverse | mouse | tcgtttggctgggataactc |
| egr2 | forward | mouse | agcctgaactggaccacct |
|  | reverse | mouse | cctgtacagcccgaataagg |
| egr3 | forward | mouse | tcggtagcccattacaatcag |
|  | reverse | mouse | ccgatgtccatcacattctct |
| egr4 | forward | mouse | agggaccaaggtcgagga |
|  | reverse | mouse | gcccctgcctggtagagt |
| ctgf | forward | mouse | tgacctggaggaaaacattaaga |
|  | reverse | mouse | agccctgtatgtcttcacactg |
| fhl2 | forward | mouse | agaaaaccatcatgccaggt |
|  | reverse | mouse | acaggtgaagcaggtctcgt |
| mall | forward | mouse | cccctttgccttcttcctac |
|  | reverse | mouse | ggggtatgcgacgtgtgt |
| nr4a3 | forward | mouse | tgcagagcctgaaccttgat |
|  | reverse | mouse | tggttcttttaacccatgtcg |
| papss2 | forward | mouse | gggggaccgagaagagaata |
|  | reverse | mouse | ttcgggcattctcacgat |
| phlda1 | forward | mouse | cacctccaactctgcctga |
|  | reverse | mouse | tgttggttttgatccaagtga |
| polq | forward | mouse | cctcctgtcatgtggcatc |
|  | reverse | mouse | cttagaatctgggtcgagaagc |
| plch1 | forward | mouse | cgcagaaaagtcaggcaaat |
|  | reverse | mouse | aaatgtcaaggttccctgattc |
| rasd1 | forward | mouse | tcagcaaactggaaactttgg |
|  | reverse | mouse | cacttggctatgattgaacacc |
| rbm6 | forward | mouse | agacactgacactagtagcaaaggag |
|  | reverse | mouse | ctcatccgcccttcaatct |
| slc1a2 | forward | mouse | agctgagagaatggtcagtgc |
|  | reverse | mouse | ttcggtgctttggctcat |
| tnfrsf11b | forward | mouse | gtgctcctggcacctaccta |
|  | reverse | mouse | agggcaagggacacacaat |
| ttc6 | forward | mouse | tctgggttatctggtccttca |
|  | reverse | mouse | gggatggtggacatcttgac |
| ttll5 | forward | mouse | ttccacatcctccctcagac |
|  | reverse | mouse | cctcggtcctttgaatatgaat |
| zmynd8 | forward | mouse | ggaaataaaaaccgagcagga |
|  | reverse | mouse | ctgtagagacaggatcttccttaggt |
| zic4 | forward | mouse | acaaaacccacaaaaccattg |
|  | reverse | mouse | acggccttcccacatctac |
| EGR1 | forward | human | agccctacgagcacctgac |
|  | reverse | human | ggtttggctggggtaactg |
| EGR2 | forward | human | ttgaccagatgaacggagtg |
|  | reverse | human | tggtttctaggtgcagagacg |
| EGR3 | forward | human | caatctgtaccccgaggaga |
|  | reverse | human | cagaccgatgtccattacattc |
| EGR4 | forward | human | gtccactgaaggctgttgc |
|  | reverse | human | gcccagctcaagaagtcg |
| CTGF | forward | human | gcctcctgcaggctagaga |
|  | reverse | human | gatgcactttttgcccttct |
| FHL2 | forward | human | ggagttggggagactgtgg |
|  | reverse | human | tccgtcccaaagtcaaaatg |
| MALL | forward | human | ctccttgatgttcctgttgtctt |
|  | reverse | human | cagtggtcccgtggtacag |
| NR4A3 | forward | human | ccgctcctcctacactctca |
|  | reverse | human | tggacgcagggcatatct |
| PAPSS2 | forward | human | ggggatcaagaagcaaaaga |
|  | reverse | human | ccccttgttccaaccactt |
| PHLDA1 | forward | human | cctccaactctgcctgaaag |
|  | reverse | human | tcgtcccacttcctcaagtc |
| POLQ | forward | human | cttggtcgctgcctgaag |
|  | reverse | human | cgtagtcaggaactgtaggcttg |
| PLCH1 | forward | human | tgaaggaaaagaaaactctctagca |
|  | reverse | human | tgtggatcttttatacttgctttcc |
| RASD1 | forward | human | cctctccatcctcacaggag |
|  | reverse | human | ggcaagacttggtgtcgag |
| RBM6 | forward | human | cctgctaacagaactggacctt |
|  | reverse | human | gtgtctctcttgagcatgactctt |
| SLC1A2 | forward | human | caaggatgggatgaacgtct |
|  | reverse | human | catcagcttggcctgatctc |
| TNFRSF11B | forward | human | cctcctggatttggagtgg |
|  | reverse | human | ccgggcctaagaatcttgt |
| TTC6 | forward | human | ggtttagtgcacaacctacacaa |
|  | reverse | human | tcttgatttccaaaggagctatg |
| TTLL5 | forward | human | gaggatgaggaggtcataagtca |
|  | reverse | human | cggcatggaataccaaaact |
| ZMYND8 | forward | human | ttctggctccagagagacg |
|  | reverse | human | ttattactccgggaatggtca |
| ZIC4 | forward | human | gcagaaatgaggcgatcct |
|  | reverse | human | tgtatctcattttctgactttgagc |
