# Supplementary figures and images for: Alternative signaling network activation through different insulin receptor family members caused by pro-mitogenic antidiabetic insulin analogues in human mammary epithelial cells
Source: Breast Cancer Res. 2015 Jul 19;17(1):97. doi: 10.1186/s13058-015-0600-5 (PMC4506606; doi:10.1186/s13058-015-0600-5)

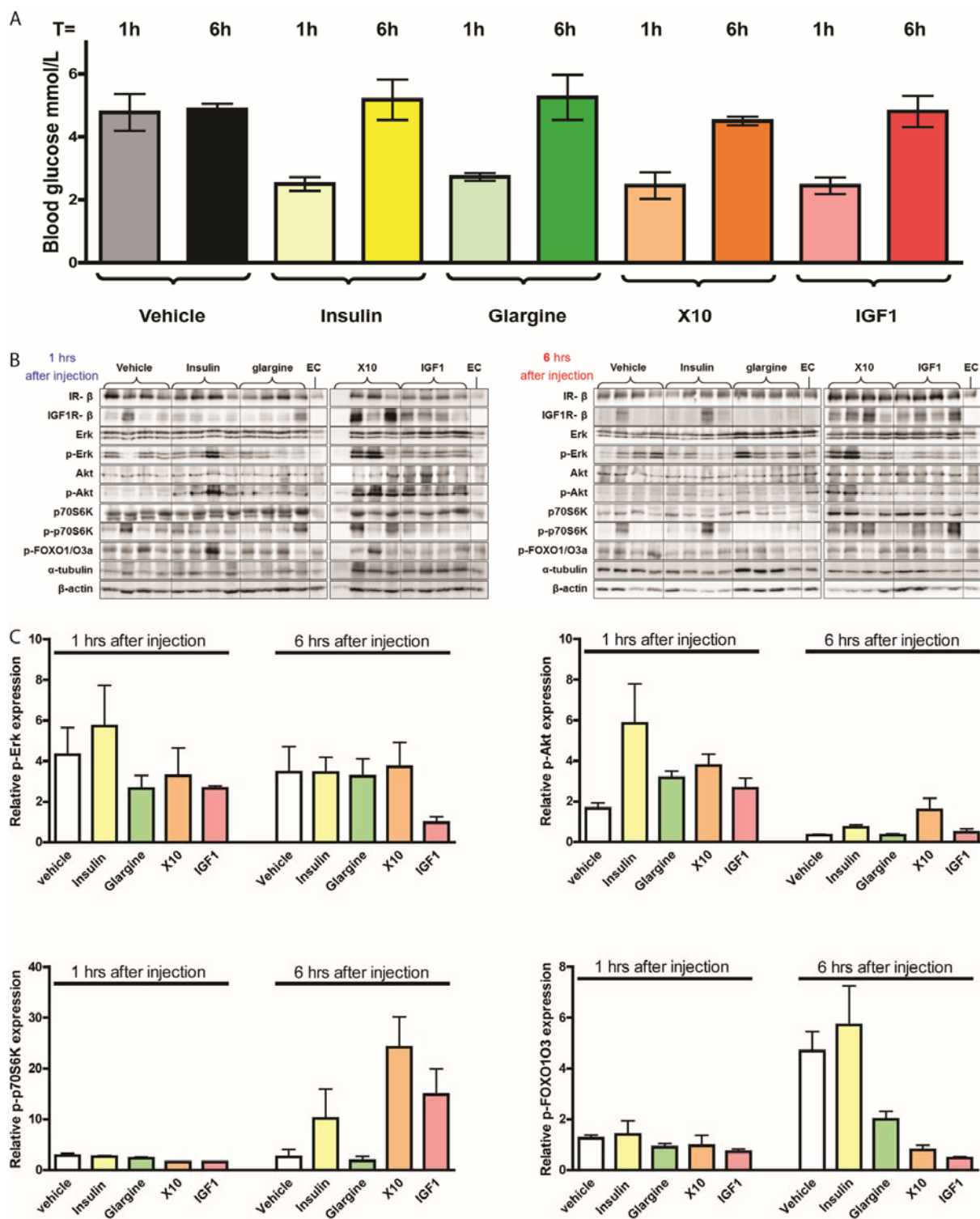

Supplement: Additional file 2: Figure S3. — Mammary gland protein levels of 40 FVB mice subcutaneously injected with insulin-like compounds. (A) The blood glucose levels (mmol/L) have been measured 1 h and 6 h after subcutaneous injections with different insulin-like molecules. (B) Western blotting of the receptors and kinases in the INSR and IGF1R pathway measured in the mammary gland tissue; samples were from 1 h (left graph) or 6 h (right graph) after the subcutaneous injections of the presented insulin-like molecules. (C) the quantification of activated kinases (Erk, Akt, p70S6K, FOXO1/O3) relative to the endogous control (EC), a sample that was loaded on every blot. N = 4. [file 13058_2015_600_MOESM2_ESM.pdf]

A

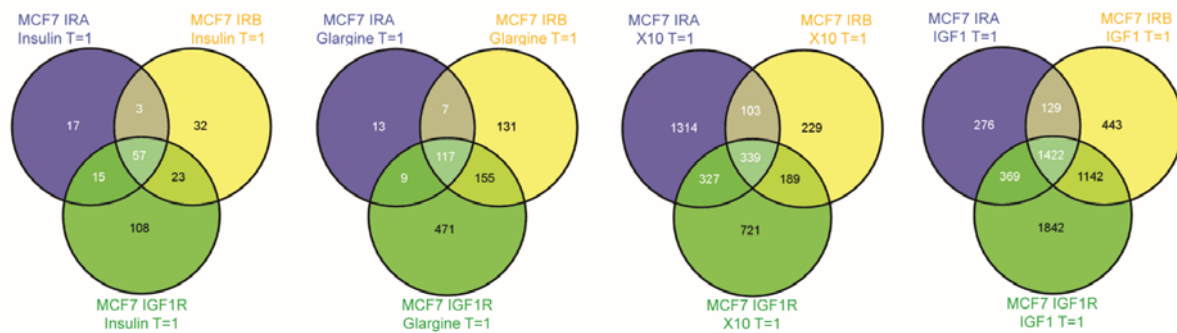

B

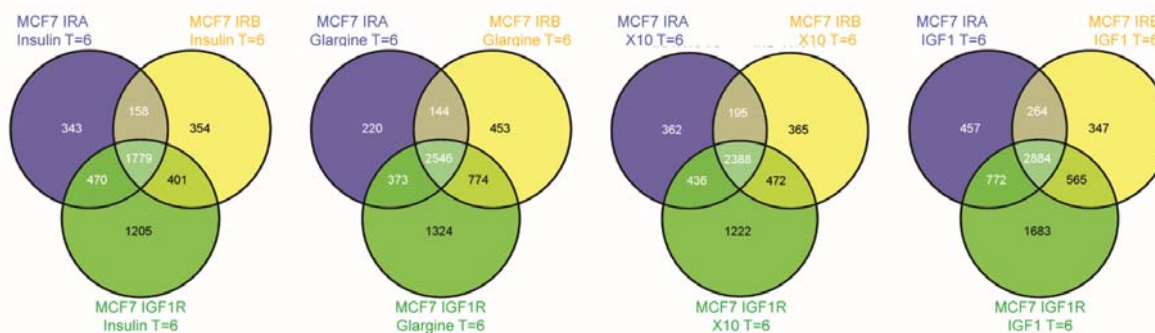

Supplement: Additional file 3: Figure S1. — Venn diagrams of the differentially expressed genes per cell line, time point and treatment showing overlap of genes between the different conditions. (A) Venn diagram of the early regulators (t = 1 h). The blue circle represents the hits in the IRA cell line, yellow the IRB cell line and green the IGF1R cell line. The first graph shows the number of hits after insulin treatment, the second graph glargine, the third X10 and the last graph shows the number of hits after IGF1 treatment. (B) The Venn diagrams of the late regulators (t = 6 h). [file 13058_2015_600_MOESM3_ESM.pdf]

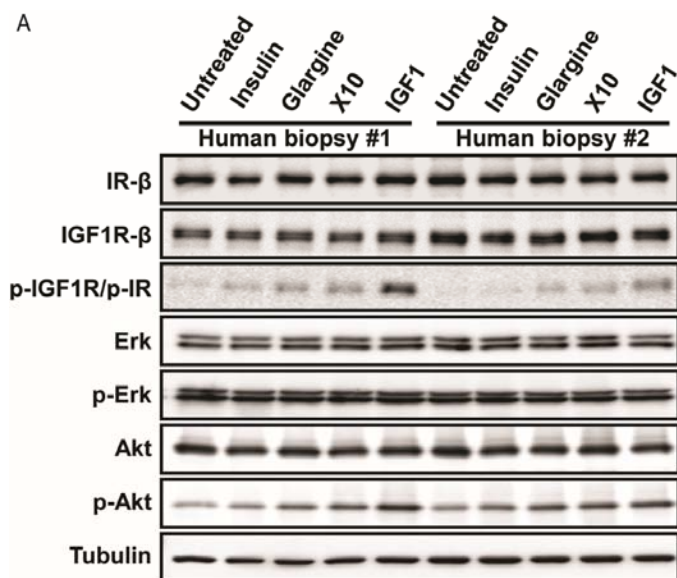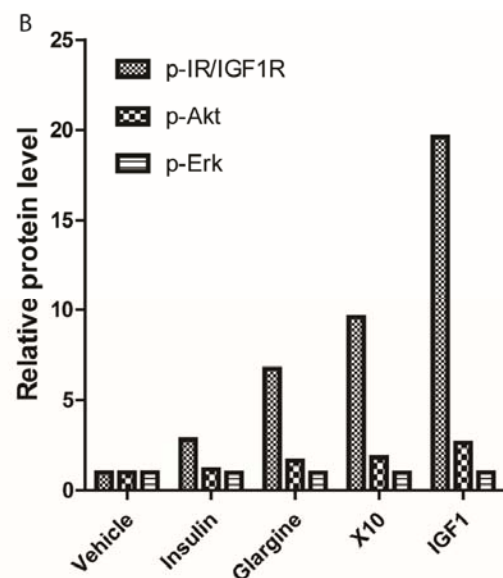

Supplement: Additional file 4: Figure S2. — Protein levels of primary human mammary cells stimulated with insulin-like compounds. (A) Primary human mammary gland cells were treated with different insulin-like molecules followed by Western blotting for various INSR and IGF1R signaling pathway components. (B) Quantification of Western blot data of the p-IGF1R/p-IR, p-Akt and p-Erk. The y-axis represents the average protein expression level compared to vehicle exposure. [file 13058_2015_600_MOESM4_ESM.pdf]
